# Diseases of Infancy and Childhood

**Published:** 1902-10

**Authors:** 


DISEASES OF INFANCY AND CHILDHOOD.—Designed for the use of students and practitioners of medicine. By Henry Koplik, M. D., attending physician to the Mount Sinai Hospital; formerly Attending Physician to the Good Samaritan Dispensary, New York; ex-President of the American Pediatric Society; Member of the Association of American Physicians and of the New York Academy of Medicine. Illustrated 165 engravings and 30 plates in color and monochrome. New York and Philadelphia. 1902. Lea Brothers & Co.

This complete, comprehensive and well-arranged work on the diseases of infancy and childhood makes its appearance at an opportune time. Although the literature on the subject has greatly increased during the last few years, but little effort seems to have been made to gather it and systematize it. Dr. Koplink is eminently qualified to prepare such a work. His many years of experience as a specialist on children's diseases and as a writer and lecturer have fitted him for the task of preparing such a work as the one under consideration.

In arrangement this volume is all that could be desired. There are fourteen chapters arranged in the following order: Infancy and Childhood, Premature Infants, The Specific Infectious Diseases, Diseases of the Mouth, Pharynx and Larynx, Diseases of the Gastro-enteric Tract, Diseases of the Heart and Pericardium, Diseases of the Nervous System, General Diseases, Diseases of the Lymph Nodes, Ductless Glands and Diseases of the Blood, Diseases of the Bones, Diseases of the Liver, Diseases of the Kidneys, and Diseases of the Skin.

The illustrations are all new and are well executed.

